# Supplementary material for: Prognosis Prediction Model After Upfront Surgery for Resectable Pancreatic Ductal Adenocarcinoma: A Multicenter Study (OS-HBP-2)
Source: Cancers (Basel). 2025 Nov 18;17(22):3694. doi: 10.3390/cancers17223694 (PMC12651716; doi:10.3390/cancers17223694)
Supplement: Supplementary file 1 [file cancers-17-03694-s001.zip › cancers-3965592-supplementary.pdf]

cancers MDPI

Scheme Digital Content S1. The prognosis prediction model for overall survival following up-front surgery in patients with resectable pancreatic ductal adenocarcinoma.

Risk calculator of the Cox regression model predicting OS	
	factor (no = 0, yes = 1)<br>(CA19-9 <40 = 0, 40-150 = 1, ≥150 = 2)
size > 2cm	1
SMV/PV contact	1
CA19-9 40/500 U/mL	0
mGPS 2	1
time after surgery (months)	60
**p (%)**	16.14257884

OS: overall survival. *p:* predictive survival probability. p (%) = 100 * exp[−0.008296 * t * exp{0.40314 * (size > 2cm) + 0.39622 * (SMV/PV contact) + 0.3962 * (CA19-9 40/500 U/mL) +0.49915 * (mGPS 2)}]. (*p* predictive survival probability, t survival time (months)).
